# Plasma EV miR-186-5p as an Early Biomarker and Regulator of IFN-α-Mediated Oxidative and β-Cell Dysfunction in Prediabetes

**DOI:** 10.3390/antiox15020150

**Published:** 2026-01-23

**Authors:** Jae-Hyung Park, Thi Nhi Nguyen, Hye Min Shim, Yun-Ui Bae, Gyeong Im Yu, Junho Kang, Eun Yeong Ha, Hochan Cho

**Affiliations:** 1 Department of Physiology, Keimyung University School of Medicine, Daegu 42601, Republic of Korea; physiopark@kmu.ac.kr (J.-H.P.); nguyenthihhiydh@gmail.com (T.N.N.); hmshim1110@gmail.com (H.M.S.); baeyunui@gmail.com (Y.-U.B.); rki0411@hanmail.net (G.I.Y.); 2 Department of Research, Keimyung University Dongsan Medical Center, Daegu 42601, Republic of Korea; junho6399@dsmc.or.kr; 3 Department of Internal Medicine, Keimyung University School of Medicine, Daegu 42601, Republic of Korea; 240012@dsmc.or.kr

**Keywords:** prediabetes, extracellular vesicles, MicroRNAs, pancreatic beta cells, interferon-alpha, oxidative stress, JAK-STAT signaling

## Abstract

Prediabetes is accompanied by early β-cell stress and oxidative imbalance before overt hyperglycemia. Circulating extracellular vesicle (EV) microRNAs (miRNAs) may capture early metabolic disturbances, but their mechanistic relevance remains unclear. Plasma EV miRNA profiles were analyzed across normoglycemia, prediabetes, and newly diagnosed type 2 diabetes, with validation in an independent cohort (n = 150). Functional studies were performed in pancreatic β-cells exposed to glucolipotoxic stress to examine miRNA regulation, IFN-α signaling, mitochondrial redox status, and insulin secretion. Six EV miRNAs, including miR-186-5p, were consistently reduced in prediabetes and correlated with glycemic and insulin resistance indices. In β-cells, glucolipotoxic stress selectively suppressed miR-186-5p, leading to derepression of IFNA2, activation of IFN-α–JAK/STAT signaling, increased mitochondrial ROS, impaired ATP/ADP dynamics, and reduced glucose-stimulated insulin secretion. Restoration of miR-186-5p or pharmacologic JAK inhibition mitigated these defects, and luciferase assays confirmed IFNA2 as a direct target of miR-186-5p. EV-associated miR-186-5p represents an early marker of metabolic stress in prediabetes and provides mechanistic insight into IFN-α–driven oxidative and secretory dysfunction in β-cells.

## 1. Introduction

Prediabetes represents an intermediate metabolic state between normoglycemia and overt type 2 diabetes mellitus (T2DM) and is characterized by impaired fasting glucose and/or impaired glucose tolerance [1,2]. Although clinically categorized as a reversible condition, accumulating evidence indicates that substantial metabolic deterioration has already occurred by the prediabetic stage, including early pancreatic β-cell dysfunction, low-grade inflammation, and mitochondrial stress [3]. Importantly, declines in β-cell secretory capacity begin well before the development of hyperglycemia detectable by conventional clinical markers such as fasting blood glucose or HbA1c [4,5,6]. Therefore, it is necessary to identify sensitive and non-invasive biomarkers capable of detecting early β-cell impairment during prediabetes, when therapeutic interventions may have the greatest potential to prevent or delay disease progression.

Extracellular vesicle (EV)–derived microRNAs (miRNAs) have emerged as promising candidates for biomarker discovery in metabolic diseases [7,8,9]. EV miRNAs circulate in a highly stable form, resist enzymatic degradation, and reflect pathophysiological changes in multiple tissues, including pancreatic islets, liver, adipose tissue, and skeletal muscle [10,11]. Several studies have shown that EV miRNA signatures are altered in T2DM and obesity, with some changes detectable even before overt metabolic disease develops [8,12,13]. Despite this potential, most studies have focused on identifying circulating EV miRNA biomarkers without determining whether these alterations directly correspond to underlying dysfunction in β-cells, the central cell type responsible for insulin production and glucose homeostasis. Consequently, the mechanistic relevance of EV miRNA dysregulation to early β-cell pathology remains poorly understood.

Pancreatic β-cells are uniquely vulnerable to metabolic and inflammatory stressors due to their limited antioxidant capacity and reliance on tightly regulated mitochondrial ATP production to support glucose-stimulated insulin secretion [14,15,16]. Excessive glucose and lipid exposure induce mitochondrial dysfunction, increase reactive oxygen species (ROS), and impair ATP generation, ultimately reducing insulin secretory responses [17,18,19]. In addition, recent work has highlighted the role of innate immune activation—particularly type I interferon (IFN-α) signaling—in β-cell stress responses [20,21]. IFN-α promotes STAT1 phosphorylation and the induction of interferon-stimulated genes (ISGs), contributing to oxidative stress, impaired insulin secretion, and β-cell senescence–like phenotypes [20,22,23,24]. However, the upstream regulators that trigger IFN-α activation and mitochondrial stress in β-cells during prediabetes remain unclear.

Given the early shifts in circulating EV miRNAs during metabolic dysregulation [7,10] and the central role of β-cell mitochondrial and immune stress in the initiation of diabetes [25,26], understanding the link between EV miRNA changes and β-cell functional decline is of critical importance. Yet whether specific EV miRNAs altered in prediabetes directly regulate β-cell innate immune signaling, mitochondrial ROS production, or insulin secretory capacity has not been established [13,27,28]. This represents a major knowledge gap at the interface of biomarker research and β-cell biology.

In this study, we sought to determine whether plasma-derived EV miRNAs are altered at the prediabetic stage and to identify miRNA candidates that directly contribute to early β-cell stress responses. Specifically, we investigated whether EV miRNAs dysregulated in prediabetes correspond to functional impairments in pancreatic β-cells, including activation of IFN-α signaling, mitochondrial oxidative stress, and defective insulin secretion. By integrating EV miRNA profiling, validation in an independent human cohort, and mechanistic experiments in β-cells, we demonstrate that miR-186-5p is substantially reduced in plasma-derived EVs during prediabetes and acts as a direct regulator of IFN-α–mediated β-cell dysfunction under diabetic stress. These findings establish miR-186-5p as both a potential early circulating biomarker and a mechanistic driver of β-cell vulnerability during the transition from normoglycemia to prediabetes.

## 2. Materials and Methods

### 2.1. Cohort Enrollment and Clinical Parameter Assessment

Two independent cohorts were recruited from Keimyung University Dongsan Medical Center between 2021 and 2024. The screening cohort included 20 subjects per group with normal blood glucose, prediabetes, or newly diagnosed type 2 diabetes mellitus (T2DM). For validation, an independent cohort of 150 subjects (n = 50 per group) was prospectively enrolled. Prediabetes and T2DM were defined according to the American Diabetes Association criteria [29]. Fasting blood samples were collected after an overnight fast. Insulin resistance was assessed by the homeostasis model assessment of insulin resistance (HOMA-IR). All participants provided written informed consent, and the study was approved by the Institutional Review Board of Keimyung University Dongsan Medical Center (IRB No. 2021-09-030, approved on 1 October 2021).

### 2.2. Plasma Collection and EV Isolation

Peripheral blood was collected in EDTA tubes. ExoQuick precipitation reagents (System Biosciences, Palo Alto, CA, USA) were then used according to the manufacturer’s protocol to isolate plasma-derived extracellular vesicles (EVs). EV pellets were resuspended in PBS and stored at −80 °C until analysis. Protein concentration of EV preparations was measured using the BCA assay (Thermo Fisher Scientific, Waltham, MA, USA).

### 2.3. Characterization of Extracellular Vesicles

Transmission electron microscopy (TEM) was performed by placing EV suspensions onto carbon-coated copper grids, staining with 2% uranyl acetate, and imaging using a Hitachi H-7000B microscope (Tokyo, Japan). Dynamic light scattering (DLS) was conducted using a Zetasizer Nano-ZS (Malvern Panalytical, Malvern, UK) to measure hydrodynamic particle size. For Western blotting, EV lysates were resolved by SDS-PAGE and probed with antibodies against CD9, TSG101 and GM130 (Abcam, Cambridge, MA, USA). The mean EV protein yield was 6.5 ± 0.9 μg/mL plasma. CD63 levels were quantified using an ExoELISA-ULTRA CD63 kit (System Biosciences).

### 2.4. Small RNA Sequencing and Candidate Selection

Small RNA libraries were generated from EV-derived RNA obtained from plasma samples using the SMARTer smRNA-Seq Kit for Illumina (Takara Bio, Shiga, Japan). Library concentration and integrity were evaluated by KAPA-based quantitative PCR and Agilent (Santa Clara, CA, USA) TapeStation analysis, respectively. Sequencing and initial bioinformatic preprocessing were performed by a commercial service provider (Macrogen, Seoul, Korea). For downstream analysis, miRNA identification was carried out using the miRDeep2 pipeline, enabling detection of both annotated and candidate novel miRNAs. Reads with unique genomic alignment were further annotated by cross-referencing miRBase (v21) and the Rfam (v9.1) database to discriminate miRNAs from other small non-coding RNA species. Differential expression analysis was performed between groups, and *p*-values were adjusted for multiple testing using the Benjamini–Hochberg false discovery rate (FDR) correction. miRNAs exhibiting an absolute fold change greater than 2.0 and an FDR-adjusted *p*-value < 0.05 were considered significantly differentially expressed and selected as candidate EV miRNAs for subsequent validation.

### 2.5. Validation of Plasma-Derived EV miRNAs by Quantitative Real-Time PCR (qRT-PCR)

EV-associated RNA isolated from plasma was converted to complementary DNA using the TaqMan™ MicroRNA Reverse Transcription Kit (Applied Biosystems, Foster City, CA, USA). EV miRNA levels were subsequently measured with sequence-specific TaqMan™ MicroRNA Assays (Cat. No. 4427975; Applied Biosystems), and relative expression was normalized to U6 small nuclear RNA. For analysis of cellular transcripts, total RNA was extracted from cultured cells using the RNeasy Mini Kit (Qiagen, Valencia, CA, USA). An equal amount of RNA (500 ng) was then subjected to reverse transcription with the High-Capacity cDNA Reverse Transcription Kit (Applied Biosystems) to generate cDNA for downstream mRNA quantification. Expression levels of IFNA2 (Mm00833961_s1), ISG15 (Mm01705338_s1), OAS1 (Mm04209919_m1), and MX1 (Mm00487796_m1) were quantified using TaqMan™ Gene Expression Assays (Applied Biosystems). Total RNA from MIN6 cells and culture supernatant–derived EVs was isolated using the miRNeasy Mini Kit and the Serum/Plasma Kit (Qiagen, Venlo, The Netherlands), respectively. qRT-PCR was conducted using TaqMan assays for miR-186-5p and miR-375. U6 snRNA (cellular) and cel-miR-39 (EVs) served as normalization controls. Relative transcript abundance was determined by comparing Ct values using a ΔΔCt-based approach, with U6 serving as the internal reference for miRNAs and GAPDH for mRNA assays.

### 2.6. Cell Culture and Treatments

MIN6 mouse pancreatic β-cells were cultured in DMEM supplemented with 10% EV-depleted FBS. EV-depleted FBS was prepared to minimize contamination from bovine EVs. Briefly, heat-inactivated FBS was diluted in PBS and ultracentrifuged at 100,000× *g* for 16 h at 4 °C using a fixed-angle rotor. The supernatant was carefully collected without disturbing the pellet, passed through a 0.22 µm filter, and aliquoted for use as EV-depleted FBS. To induce diabetic stress, MIN6 cells were treated with high glucose (25 mM) and palmitate (0.5 mM; HG+PA) for 24–48 h. Ruxolitinib (1 μM; Cayman Chemical, Ann Arbor, MI, USA) was used to inhibit JAK signaling. Unless otherwise specified, gene expression and signaling analyses were performed after 24 h of HG+PA exposure, whereas mitochondrial function and insulin secretion assays were conducted after 48 h. To elevate intracellular miR-186-5p levels, cells received the synthetic miR-186-5p mimic (mirVana, Thermo Fisher Scientific) delivered via Lipofectamine RNAiMAX. The mimic was used at a working concentration of 50 nM for all experiments. A mock (RNAiMAX-only) control and a negative control (NC) mimic were included in all experiments. The RNAiMAX-to-miRNA ratio followed the manufacturer’s recommendations.

### 2.7. Measurements of Mitochondrial ROS and Insulin Secretion

Mitochondrial superoxide levels were assessed using MitoSOX Red (Thermo Fisher Scientific). MIN6 cells were incubated with 5 µM MitoSOX for 10 min at 37 °C, washed with HBSS, and imaged using a confocal laser microscope (LSM 5 EXCITER; Carl Zeiss, Jena, Germany). Fluorescence intensity was quantified from 5 random fields per sample using ImageJ software version 1.53c. Mean fluorescence intensity was calculated after background subtraction, and values were normalized to cell number. To measure GSIS, MIN6 cells were pre-incubated in Krebs–Ringer bicarbonate (KRB) buffer containing 2.8 mM glucose for 2 h, followed by stimulation with 20 mM glucose for 30 min. Insulin concentrations in supernatants were determined using a mouse insulin ELISA kit (Mercodia, Uppsala, Sweden) according to the manufacturer’s instructions. Insulin secretion was normalized to total cellular protein content. Glucose-stimulated ATP/ADP ratio was quantified using the ADP/ATP Ratio Assay Kit (Sigma-Aldrich, Burlington, MA, USA, Cat# MAK135). MIN6 cells were incubated with low glucose (2.8 mM) KRB for 2 h and then stimulated with 20 mM glucose for 30 min. Cells were lysed, and luminescence was measured using a microplate luminometer following the manufacturer’s protocol. The ATP/ADP ratio was calculated based on sequential luminescence readings for ATP and ADP in each well.

### 2.8. Western Blot Analysis

Equal amounts of protein from each sample were electrophoresed on SDS-PAGE gels and transferred to nitrocellulose membranes. The membranes were probed with specific antibodies as follows: anti-STAT1 (Abcam), anti-phospho-STAT1 (Abcam), and anti-GAPDH (Sigma–Aldrich). The membranes were incubated with horseradish peroxidase-coupled secondary antibody (Sigma–Aldrich). Following washing with TBS-T, the bound antibody was detected by enhanced chemiluminescence (Amersham, UK). Protein levels of IFN-α in culture supernatants were measured via ELISA (Thermo Fisher Scientific).

### 2.9. Luciferase Reporter Assay

A fragment of the mouse IFNA2 3′UTR—either in its native form or carrying mutations in the predicted miR-186-5p binding region—was inserted into the pmirGLO vector downstream of the firefly luciferase cassette (Promega, Madison, WI, USA). A fragment of the mouse IFNA2 3′UTR harboring the miR-186-5p seed-matching region (5′-UUCUUU-3′) was amplified and inserted into pmirGLO to generate the wild-type construct (pmirGLO/IFNA2-WT-Luc). A mutant reporter (pmirGLO/IFNA2-Mut-Luc) was created by substituting three nucleotides within the seed-matching sequence to disrupt miR-186-5p binding. For the reporter assay, HEK293 cells were transfected with 200 ng of the luciferase constructs together with 50 nM miR-186-5p mimic or a negative control oligonucleotide using Lipofectamine RNAiMAX (Thermo Fisher Scientific). After 24 h, the Dual-Luciferase Reporter Assay System (Promega) was used to quantify luciferase activity.

### 2.10. Statistical Analysis

Normally distributed variables were analyzed using one-way ANOVA with Tukey’s post hoc test. Differences between two groups were assessed using two-tailed Student’s *t*-tests. Associations between EV miRNA levels and clinical variables were examined using Pearson’s correlation analysis and logistic regression modeling. Clinical characteristics are reported as mean values with standard deviations, whereas data obtained from in vitro experiments are presented as mean values with standard errors of the mean. Statistical significance was set at *p* < 0.05. Analyses were performed using SPSS version 29.

## 3. Results

### 3.1. Screening Analysis of Subjects with Normoglycemia, Prediabetes, and T2DM

Table 1 summarizes the clinical characteristics of subjects with normoglycemia, prediabetes, and newly diagnosed T2DM. As expected, fasting blood glucose, HbA1c, fasting plasma insulin, and HOMA-IR were progressively elevated across the three groups. In contrast, sex distribution, BMI, waist circumference, creatinine, eGFR, LDL cholesterol, AST, ALT, and blood pressure showed no differences among the groups, indicating that the metabolic deterioration was primarily reflected in glycemic and insulin-resistance parameters.

### 3.2. Plasma-Derived EV miRNA Expression in Prediabetes and T2DM

**Figure 1:**
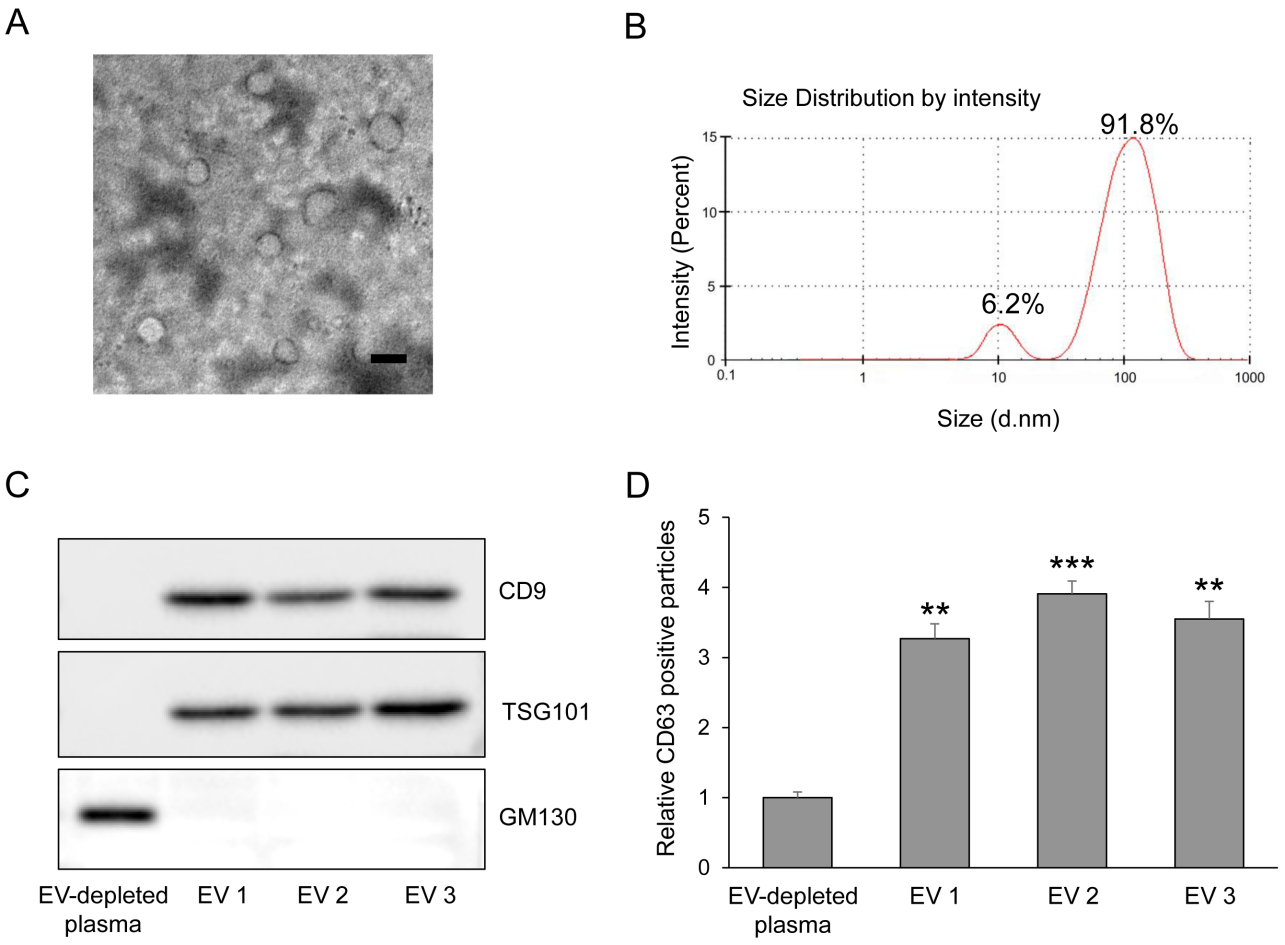
Characterization of plasma-derived extracellular vesicles. (**A**) Transmission electron microscopy (TEM) images showing round, membrane-bound morphology of purified plasma EVs. Scale bar = 200 nm. (**B**) Dynamic Light Scattering (DLS) profile of plasma-derived EVs obtained using the Nano-ZS Zetasizer, illustrating the hydrodynamic size distribution of EV particles. (**C**) Western blot analysis of EV-enriched markers (TSG101 and CD9) and the Golgi marker GM130, confirming enrichment of EV proteins and minimal contamination from cellular organelles. (**D**) Quantification of CD63 expression in EV-depleted plasma and plasma-derived EV fractions using ELISA, confirming successful enrichment of CD63-positive EVs (n = 5). The values represent the mean ± SEM. ** *p* < 0.01 and *** *p* < 0.001 vs. control. EV, extracellular vesicle.

**Figure 2:**
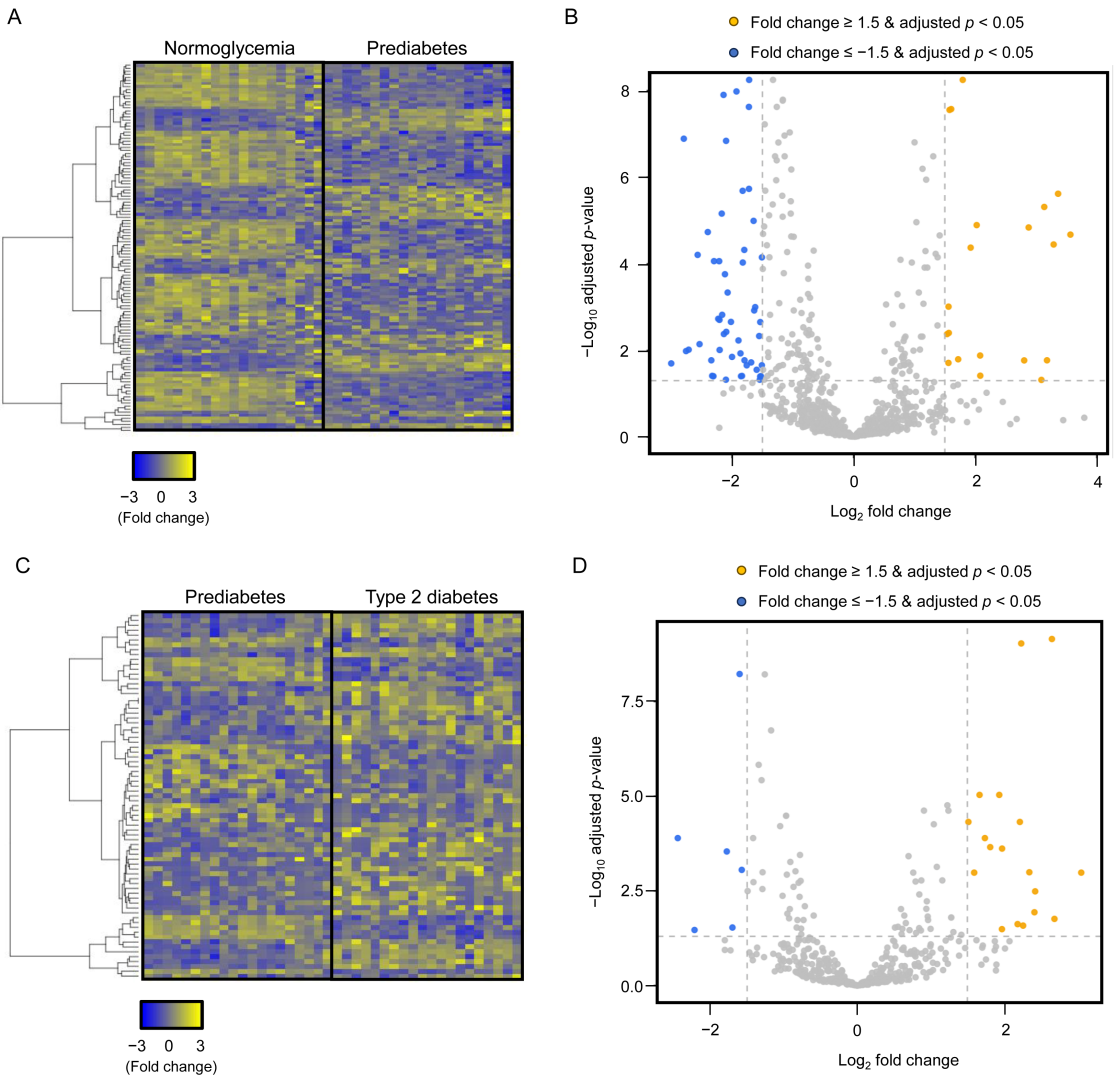
Differential expression of plasma-derived EV miRNAs across glycemic groups. (**A**) Heatmap showing z-score–normalized expression patterns of plasma-derived EV miRNAs in subjects with normoglycemia and prediabetes. Yellow and blue indicate relative upregulation and downregulation, respectively. (**B**) Volcano plot illustrating differentially expressed EV miRNAs in prediabetes compared with normoglycemia. Statistical significance was determined using false discovery rate (FDR)–adjusted *p*-values. miRNAs with an absolute fold change > 2 and FDR < 0.05 are highlighted. (**C**) Heatmap showing z-score–normalized expression patterns of EV miRNAs in subjects with prediabetes and T2DM. (**D**) Volcano plot illustrating differentially expressed EV miRNAs in T2DM compared with prediabetes using FDR-adjusted *p*-values (|fold change| > 2, FDR < 0.05). EV, extracellular vesicle; miRNA, microRNA; T2DM, type 2 diabetes mellitus.

Plasma-derived EV miRNA profiling was performed in the screening cohort using paired plasma samples from subjects with normoglycemia, prediabetes, and T2DM. Following EV isolation, the identity and purity of plasma-derived EVs were confirmed using standard characterization methods. Transmission electron microscopy revealed round, membrane-bound vesicular structures with an average diameter of 160.3 ± 29.8 nm (Figure 1A). Dynamic light scattering analysis demonstrated a median hydrodynamic diameter of 173.6 ± 61.5 nm, consistent with the expected size range of plasma-derived EVs (Figure 1B). Western blot analysis showed robust expression of the EV-enriched marker proteins CD9 and TSG101, while the lipoprotein contamination marker APOA1 was minimally detected, indicating high EV purity (Figure 1C). In addition, CD63 levels were markedly higher in isolated EV fractions compared with EV-depleted plasma, as determined by ELISA (Figure 1D). Small RNA sequencing analysis demonstrated clear alterations in plasma-derived EV miRNA profiles across the three glycemic groups (Figure 2A,C). To account for multiple comparisons, differential expression analysis was performed using false discovery rate (FDR) correction. Following FDR adjustment, comparison between normoglycemia and prediabetes identified 39 miRNAs that were significantly upregulated and 81 miRNAs that were significantly downregulated in prediabetes (Figure 2B). Likewise, comparison between prediabetes and T2DM revealed 46 upregulated and 28 downregulated EV miRNAs (Figure 2D). For transparency, volcano plots generated using unadjusted *p*-values are provided as Supplementary Figure S1. Based on an absolute fold change greater than 2.0 and an FDR-adjusted *p*-value < 0.05, 12 EV miRNAs showing the most robust and consistent differential expression between groups were selected as candidates for subsequent validation (Table 2).

### 3.3. Validation of Selected EV miRNAs in an Independent Cohort

**Figure 3:**
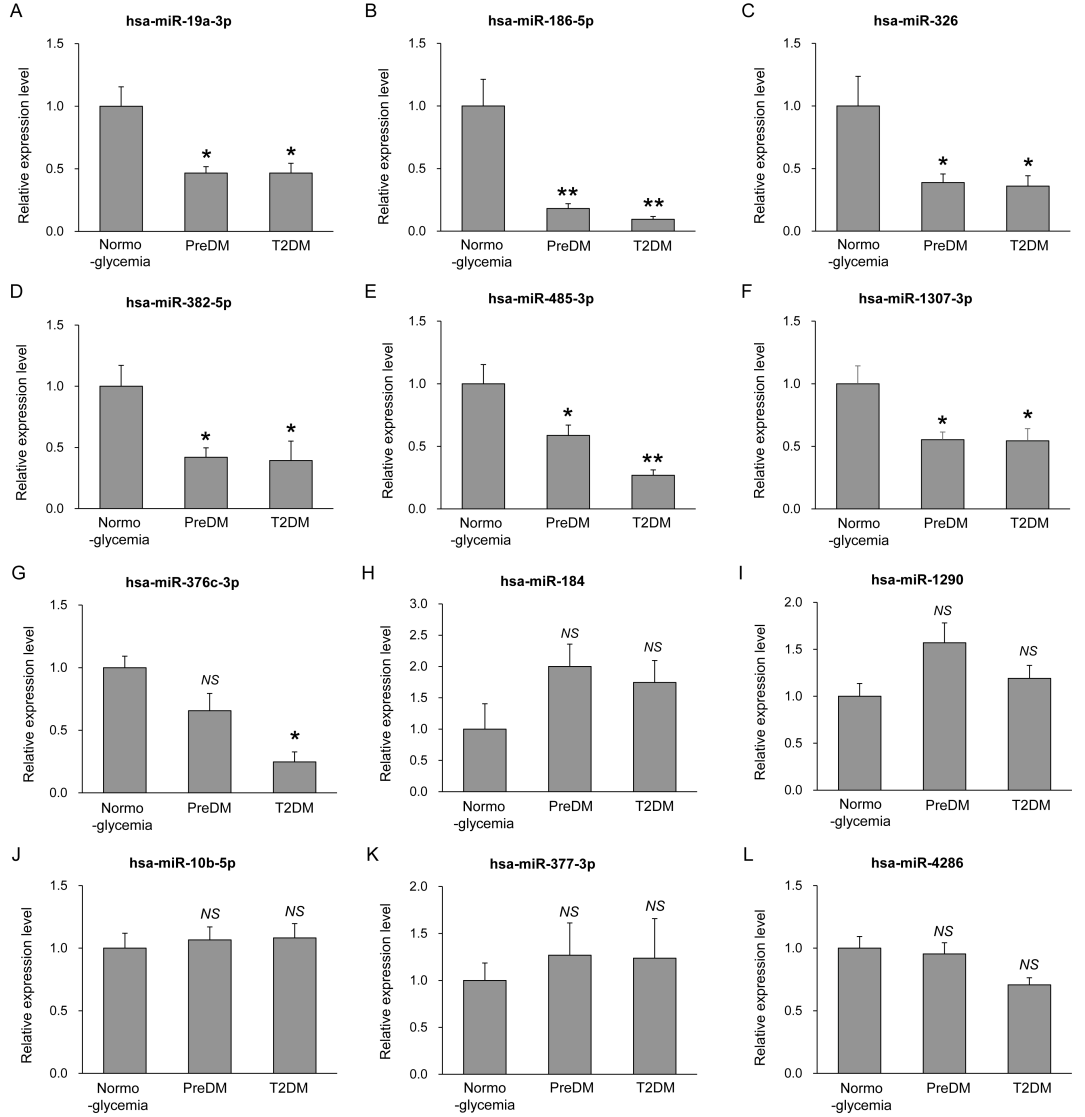
Validation of selected EV miRNAs in an independent cohort. Expression levels of plasma-derived EV miRNAs in subjects with normoglycemia (n = 50), prediabetes (n = 50), and T2DM (n = 50). Expression levels of miR-19a-3p (**A**), miR-186-5p (**B**), miR-326 (**C**), miR-382-5p (**D**), miR-485-3p (**E**), miR-1307-3p (**F**), miR-376c-3p (**G**), miR-184 (**H**), miR-1290 (**I**), miR-10b-5p (**J**), miR-377-3p (**K**), and miR-4286 (**L**) were determined by qRT-PCR analysis. Results are shown as mean ± SEM (n = 50). * *p* < 0.05 and ** *p* < 0.01 compared to normoglycemia; NS, not statistically significant.

To validate the 12 plasma-derived EV miRNAs identified in the screening analysis, we examined a newly recruited independent cohort of 150 subjects (50 normoglycemia, 50 prediabetes, and 50 T2DM) (Table 3). As observed in the screening cohort, fasting blood glucose, HbA1c, fasting insulin, and HOMA-IR were significantly elevated across the metabolic spectrum, whereas sex, BMI, waist circumference, creatinine, eGFR, LDL cholesterol, AST, ALT, and blood pressure did not differ among the groups. EV miRNA levels were quantified using qRT-PCR (Figure 3). Among the 12 candidates, six miRNAs—miR-19a-3p, miR-186-5p, miR-326, miR-382-5p, miR-485-3p, and miR-1307-3p—were significantly downregulated in prediabetes compared with normoglycemia and remained reduced in T2DM (Figure 3A–F). In contrast, miR-376c-3p showed a significant reduction only in T2DM, but not in prediabetes, relative to normoglycemia (Figure 3G). The other miRNAs showed no significant changes in prediabetes or T2DM compared to normal glycemia (Figure 3H–L).

### 3.4. Association Between Plasma-Derived EV miRNAs and Metabolic Parameters

To evaluate whether alterations in plasma-derived EV miRNAs were associated with metabolic deterioration, we examined correlations between the six validated miRNAs and key clinical parameters, including fasting blood glucose, HbA1c, fasting insulin, and HOMA-IR, using Pearson correlation analysis. As shown in Table 4, all six miRNAs exhibited significant negative correlations with these metabolic indices, indicating that lower EV miRNA levels were associated with greater impairment of glucose metabolism. To further assess their predictive value for prediabetes, univariate and multivariate logistic regression analyses were performed. Among the six candidates, only miR-186-5p remained an independent predictor of prediabetes after adjustment for relevant clinical variables (Table 5), supporting its potential utility as an early EV-based biomarker of metabolic dysregulation.

**Figure 4:**
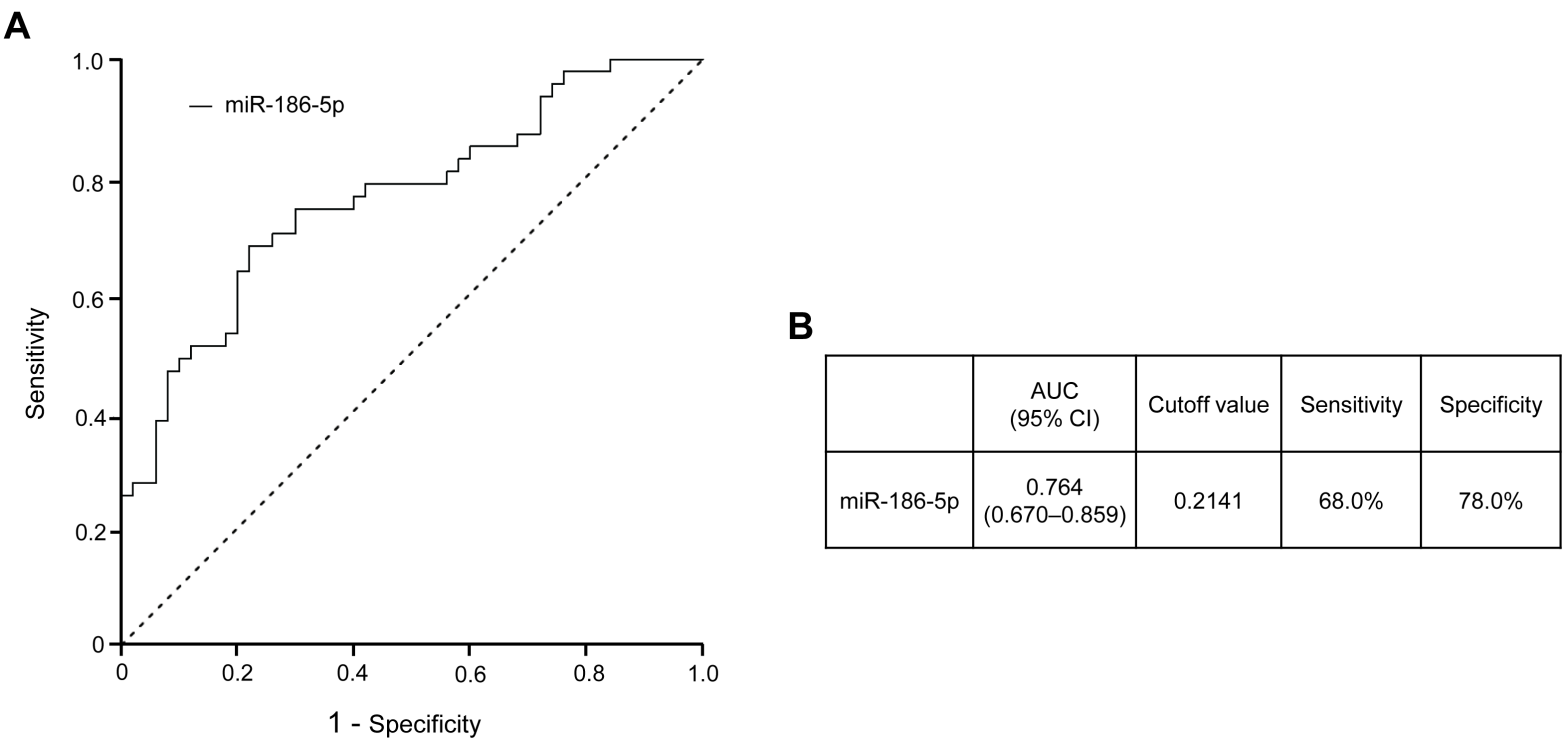
Diagnostic performance of plasma-derived EV miR-186-5p for prediabetes. (**A**) Receiver operating characteristic (ROC) curve of plasma EV miR-186-5p distinguishing individuals with prediabetes from those with normoglycemia. (**B**) Area under the ROC curve (AUC) with corresponding 95% confidence interval (CI), sensitivity, specificity, and optimal cutoff value for plasma EV miR-186-5p. The cutoff value was determined using the Youden index to achieve an optimal balance between sensitivity and specificity. The dash line represents the reference line for random classification (AUC = 0.5). AUC, area under the curve; CI, confidence interval.

Receiver operating characteristic (ROC) curve analysis was additionally conducted to evaluate the discriminatory performance of plasma-derived EV miR-186-5p for prediabetes (Figure 4). The ROC analysis demonstrated that miR-186-5p exhibited good diagnostic performance (AUC 280 = 0.745, 95% CI, 0.578–0.912), with the area under the curve (AUC) indicating a meaningful ability to distinguish individuals with prediabetes from normoglycemic subjects. An optimal cutoff value was estimated using the Youden index as an exploratory reference; however, this threshold should be interpreted cautiously, as the present study was not designed to establish definitive diagnostic cutoffs, and further validation in larger prospective cohorts will be required.

### 3.5. Roles of EV miRNAs in Pancreatic β-Cells Under Diabetic Conditions

**Figure 5:**
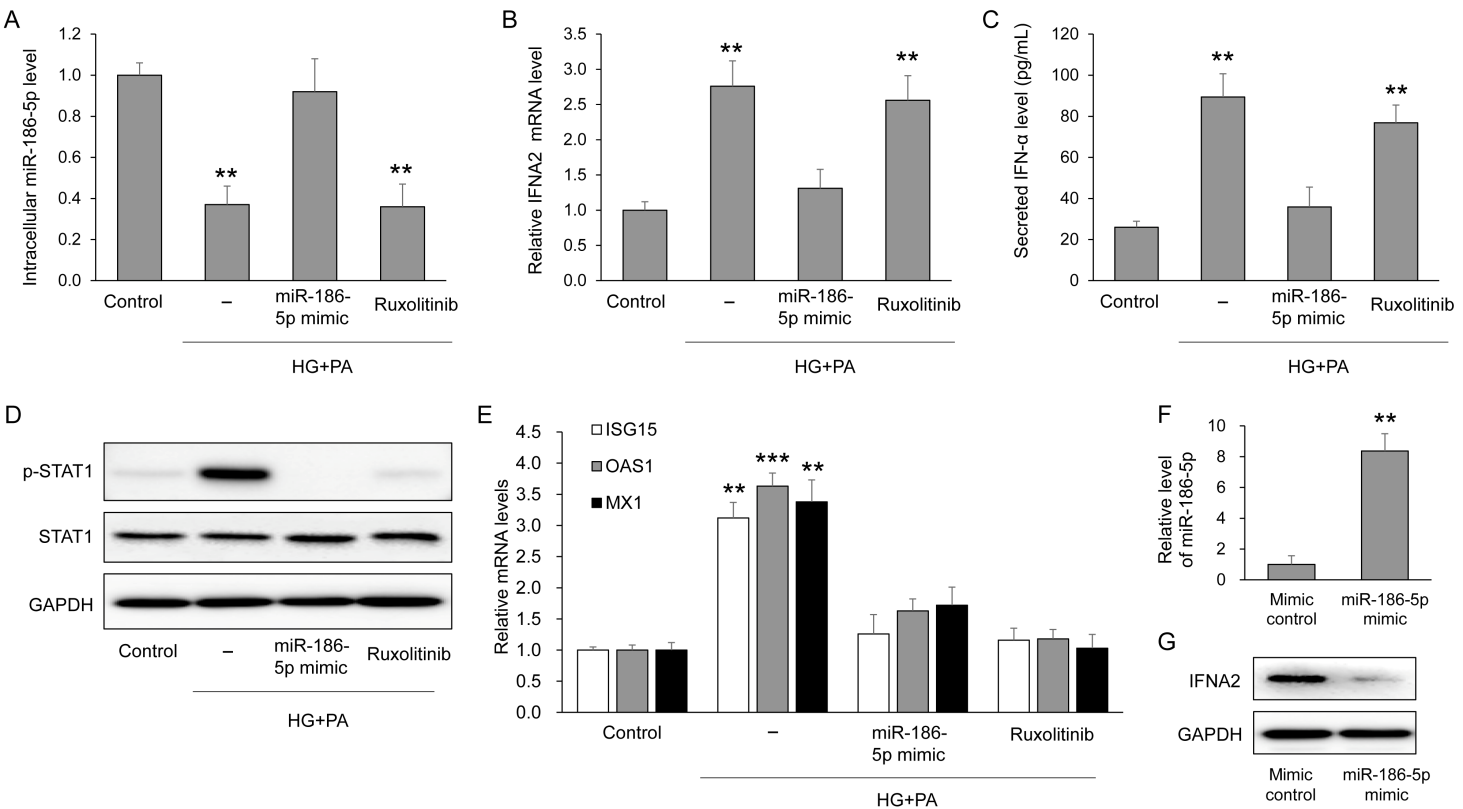
miR-186-5p regulates IFN-α signaling in β-cells under diabetic stress. MIN6 cells were exposed to high glucose (25 mM) and palmitate (0.5 mM; HG+PA) in the presence or absence of miR-186-5p mimic or the JAK inhibitor ruxolitinib. (**A**) Intracellular miR-186-5p levels following HG+PA treatment. (**B**) IFNA2 mRNA expression after HG+PA exposure. (**C**) Secreted IFN-α protein levels measured in culture supernatants. (**D**) Representative immunoblots and quantification of total STAT1 and phosphorylated STAT1 (p-STAT1). (**E**) Relative mRNA expression levels of interferon-stimulated genes (ISG15, OAS1, and MX1). (**F**) qRT-PCR validation of miR-186-5p overexpression following miR-186-5p mimic transfection under basal conditions. (**G**) IFNA2 protein expression following miR-186-5p mimic transfection. Data are presented as mean ± SEM from at least three independent experiments (n = 5). ** *p* < 0.01 and *** *p* < 0.001 vs. each corresponding control.

To determine whether the EV miRNAs altered in circulation also respond to diabetic stress within pancreatic β-cells, we examined intracellular levels of the six validated miRNAs in MIN6 cells exposed to high glucose and palmitate (HG+PA). Among these candidates, only miR-186-5p showed a significant reduction after HG+PA treatment compared with control cells (Supplementary Figure S2). Consistently, intracellular miR-186-5p levels were markedly decreased by HG+PA but were restored by transfection with a miR-186-5p mimic, whereas ruxolitinib, a JAK inhibitor, had no effect on miR-186-5p expression (Figure 5A). Successful overexpression of miR-186-5p following mimic transfection was directly confirmed by qRT-PCR analysis (Figure 5F). HG+PA treatment significantly increased IFNA2 mRNA expression, which was effectively suppressed by miR-186-5p mimic but not by ruxolitinib (Figure 5B). Consistent with these mRNA changes, IFNA2 protein expression was also markedly reduced following miR-186-5p mimic transfection (Figure 5G). Secreted IFN-α levels in the culture supernatant closely mirrored the intracellular IFNA2 expression pattern (Figure 5C). Activation of downstream signaling was evidenced by enhanced STAT1 phosphorylation under HG+PA conditions, which was normalized by either miR-186-5p mimic or ruxolitinib (Figure 5D). Furthermore, the expression of interferon-stimulated genes (ISG15, OAS1, and MX1) exhibited a similar pattern, showing induction by HG+PA and suppression by both miR-186-5p mimic and ruxolitinib (Figure 5E).

### 3.6. Effects of miR-186-5p on Mitochondrial ROS and Insulin Secretion in Pancreatic β-Cells

**Figure 6:**
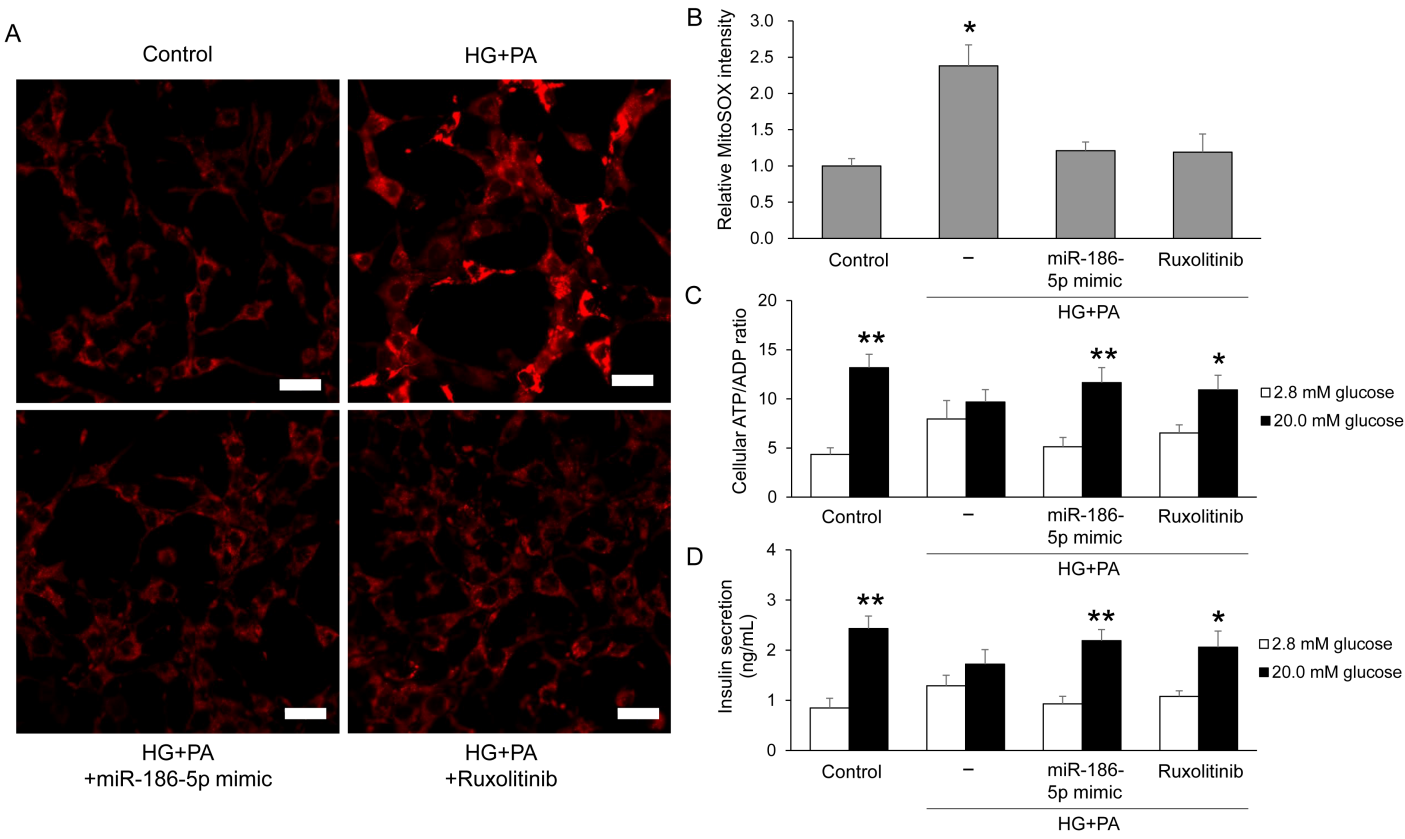
miR-186-5p restores mitochondrial function and insulin secretion in β-cells under diabetic stress. MIN6 cells were exposed to HG+PA with or without miR-186-5p mimic or ruxolitinib. (**A**) Representative MitoSOX fluorescence images showing mitochondrial superoxide production. Scale bar = 20 μm. (**B**) Quantification of MitoSOX fluorescence intensity. (**C**) Glucose-stimulated ATP/ADP ratio. (**D**) Glucose-stimulated insulin secretion measured in culture supernatants. For functional assays, cells were pre-incubated in Krebs–Ringer bicarbonate buffer containing 2.8 mM glucose for 2 h, followed by stimulation with 20 mM glucose for 30 min. Data represent mean ± SEM from at least three independent experiments (n = 5). * *p* < 0.05 and ** *p* < 0.01 vs. respective controls.

To assess whether miR-186-5p modulates mitochondrial oxidative stress in β-cells, we measured mitochondrial superoxide levels using MitoSOX fluorescence in MIN6 cells. HG+PA treatment markedly increased MitoSOX intensity compared with control cells, indicating enhanced mitochondrial ROS production. This increase was significantly attenuated by either miR-186-5p mimic or ruxolitinib (Figure 6A,B). We next evaluated β-cell functional outcomes by measuring glucose-stimulated changes in the intracellular ATP/ADP ratio and insulin secretion. Both parameters were robustly elevated in control cells upon glucose stimulation but were substantially blunted by HG+PA exposure. Importantly, these impairments were restored by miR-186-5p mimic or ruxolitinib treatment (Figure 6C,D), demonstrating that miR-186-5p plays a critical role in preserving mitochondrial function and insulin secretory capacity under diabetic stress.

### 3.7. IFNA2 as a Direct Target of miR-186-5p in Pancreatic β-Cells

**Figure 7:**
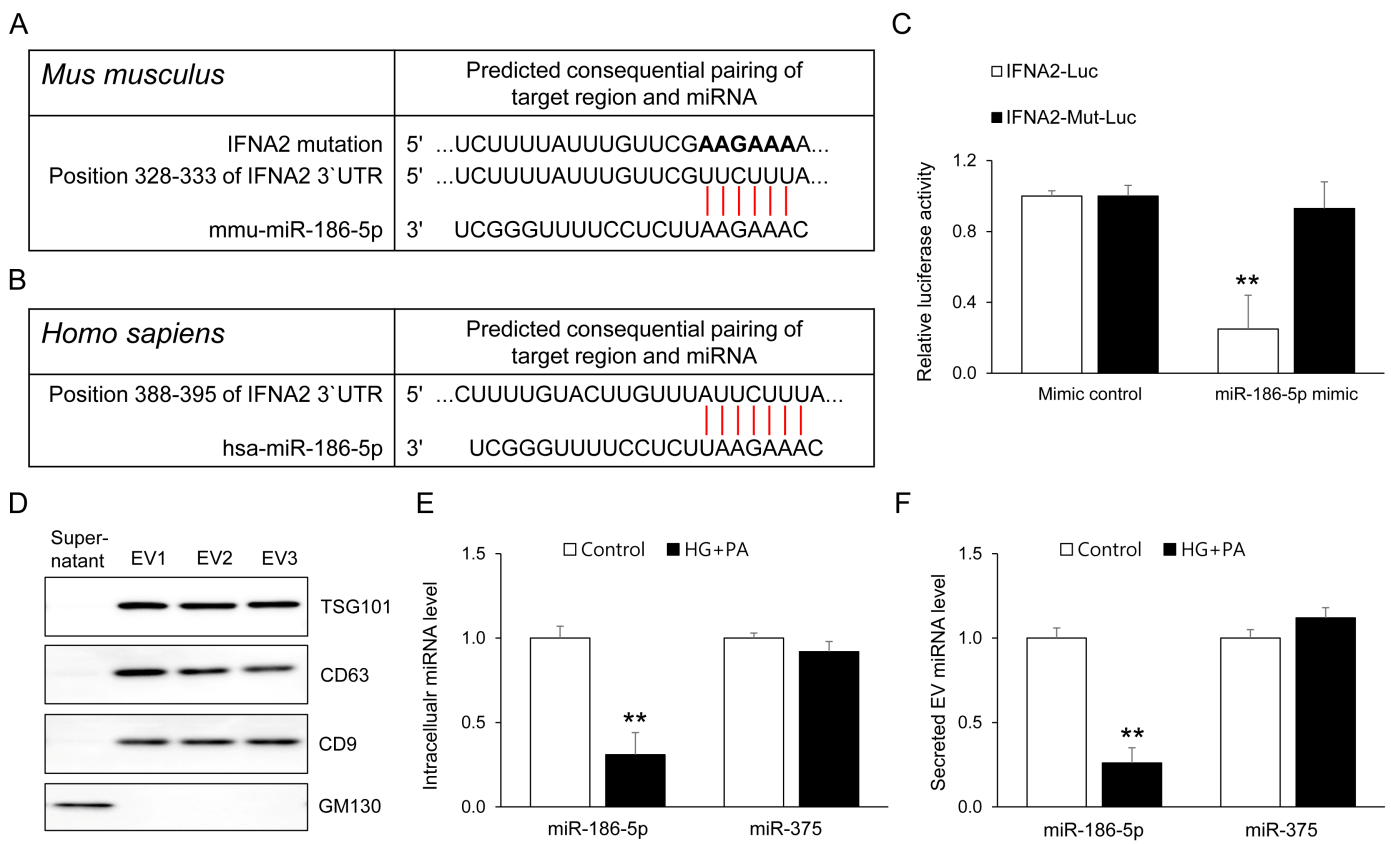
IFNA2 is a direct target of miR-186-5p and its EV export reflects β-cell stress. (**A**,**B**) TargetScan prediction of conserved miR-186-5p binding sites within the mouse and human IFNA2 3′ untranslated regions (3′UTRs). (**C**) Dual-luciferase reporter assays performed in HEK293 cells co-transfected with wild-type (WT) or mutant (Mut) IFNA2 3′UTR constructs and miR-186-5p mimic. (**D**) Western blot analysis of EV-enriched markers (CD63, CD9, TSG101) and the Golgi marker GM130 in EVs isolated from MIN6 cell supernatants. (**E**) Intracellular levels of miR-186-5p and miR-375 following HG+PA treatment. (**F**) Levels of miR-186-5p and miR-375 in EVs released into the culture supernatant. Relative miRNA expression was quantified by qRT-PCR. Data represent mean ± SEM from at least three independent experiments (n = 5). ** *p* < 0.01 vs. respective controls. Luc, luciferase; Mut, mutant.

To identify direct downstream targets of miR-186-5p relevant to β-cell stress responses, we performed in silico target prediction using TargetScan. This analysis identified IFNA2 as a conserved putative target of miR-186-5p in both mice and humans, with a predicted binding site located within the 3′ untranslated region (3′UTR) (Figure 7A,B). To experimentally validate this interaction, we measured luciferase activity in HEK293 cells co-transfected with a miR-186-5p mimic and a luciferase reporter containing the wild-type IFNA2 3′UTR. Introducing the miR-186-5p mimic led to a marked reduction in reporter activity, whereas altering the seed-binding region completely eliminated this effect (Figure 7C). These results demonstrate that miR-186-5p directly interacts with the IFNA2 3′UTR. We next examined whether β-cells themselves contribute to the reduction in circulating EV miR-186-5p observed in prediabetes. EVs were isolated from MIN6 cell supernatants, and EV identity was confirmed using established markers (Figure 7D). HG+PA treatment markedly decreased miR-186-5p expression in both intracellular and EV fractions, whereas the abundance of the β-cell–enriched miRNA miR-375 remained unchanged (Figure 7E,F). These findings suggest that β-cells may represent one biologically relevant contributor to the reduction in circulating EV miR-186-5p during prediabetes. Thus, EV miR-186-5p released from stressed β-cells mirrors intracellular miR-186-5p depletion and may partially explain the lowered plasma EV miR-186-5p levels observed in individuals with prediabetes.

## 4. Discussion

In this study, we identified plasma-derived EV miRNAs that are altered in prediabetes and newly diagnosed T2DM and demonstrated that one of these candidates, miR-186-5p, plays a mechanistic role in regulating β-cell innate immune signaling, mitochondrial function, and insulin secretion under diabetic stress. Our findings reveal a previously unrecognized association between early changes in circulating EV miRNAs and intracellular β-cell stress responses, supporting the concept that EV miR-186-5p may serve as an early, non-invasive biomarker of metabolic deterioration. In addition, our mechanistic data indicate that miR-186-5p functions as an intracellular regulator of β-cell stress pathways under glucolipotoxic conditions.

Prediabetes is now understood as a metabolic condition in which insulin resistance and early β-cell dysfunction are already established, even though alterations in conventional glycemic indices remain relatively modest [5,30,31]. Standard clinical markers, including fasting glucose and HbA1c, therefore have limited sensitivity for capturing early metabolic decline at this stage [32,33,34]. In this context, our analysis revealed distinct changes in plasma-derived EV miRNA profiles during prediabetes, with several miRNAs showing significant downregulation prior to the onset of overt T2DM. Notably, EV miR-186-5p displayed consistent reductions across both the screening and validation cohorts and remained independently associated with prediabetes, supporting its potential value as an early, non-invasive indicator of metabolic stress. Consistent with this observation, ROC curve analysis demonstrated that EV miR-186-5p provides meaningful discriminatory capacity for prediabetes, although its clinical cutoff should be considered exploratory. The intrinsic stability and tissue-informative nature of EV miRNAs further support their suitability for early disease detection [35,36].

Circulating miR-186-5p has been reported as a diagnostic or prognostic marker across multiple disease settings, including cardiovascular disorders [37] and cancer [38], and more recently in metabolic disease contexts [39]. Collectively, these studies support the view that miR-186-5p functions as a broadly stress-responsive miRNA in circulation, but they also underscore a key challenge—its circulating abundance may reflect integrated systemic perturbations rather than a single tissue source. In this context, our data align with the existing literature by demonstrating that miR-186-5p is sensitive to metabolic stress, while extending prior work in two important directions. First, we specifically profiled EV-associated miR-186-5p, a compartment that is biologically stable and may better preserve disease-related signals than total plasma miRNA [35,40]. Second, we focused on the early transition from normoglycemia to prediabetes, showing consistent downregulation in independent cohorts, which suggests potential utility for early-stage risk stratification. Moreover, by linking miR-186-5p depletion to derepression of IFNA2 and activation of IFN-α–JAK/STAT signaling in β-cells, our findings provide a mechanistic context for how a circulating EV miRNA alteration may relate to β-cell oxidative and secretory dysfunction during early metabolic deterioration [41,42,43]. Nonetheless, given the non–tissue-specific nature of miR-186-5p, future work should clarify tissue contributions to circulating EV miR-186-5p and evaluate its specificity within broader cardiometabolic and inflammatory conditions.

The current study extends the role of miR-186-5p beyond its previously established involvement in endothelial dysfunction [44,45], renal fibrosis [46,47], and inflammatory signaling [48,49], and cardiovascular pathology, including cardiomyopathy [37], by defining its functional relevance within pancreatic β-cells under metabolic stress. We demonstrate that miR-186-5p is selectively downregulated in β-cells exposed to high glucose and palmitate, indicating that miR-186-5p is intrinsically responsive to glucolipotoxic stress at the cellular level. This selective reduction supports the concept that miR-186-5p exerts a protective role in β-cells by restraining stress-induced signaling pathways. Importantly, restoration of miR-186-5p levels by mimic transfection confirms that its suppression represents a specific regulatory response to β-cell stress rather than a nonspecific or global alteration of miRNA expression.

One of the key findings of this study is the identification of IFNA2 as a direct and functionally relevant target of miR-186-5p in β-cells. Under physiological conditions, β-cells express minimal IFN-α; however, metabolic or inflammatory stress can trigger innate immune programs, promoting IFN-α production and downstream STAT1 phosphorylation [20,50,51]. Sustained activation of the IFN-α–STAT1–ISG axis has been linked to β-cell dysfunction, oxidative stress, enhanced antigen presentation, and senescence-like phenotypes [23,24,51,52,53]. In this study, luciferase assays confirmed that miR-186-5p directly binds the IFNA2 3′UTR and suppresses its expression, providing a mechanistic basis for the observation that miR-186-5p loss permits IFN-α upregulation under glucolipotoxic stress. To our knowledge, this miR-186-5p–IFNA2 regulatory relationship has not been previously described in β-cells. In this context, interferon-stimulated genes (ISG15, OAS1, and MX1) were evaluated as canonical downstream readouts of IFN-α–JAK/STAT signaling rather than as direct markers of insulin signaling. This experimental strategy was chosen to mechanistically link miR-186-5p–IFNA2 dysregulation to innate immune activation in β-cells, a process that precedes and contributes to mitochondrial dysfunction and impaired insulin secretion during early metabolic stress. Because miR-186-5p is predicted to regulate multiple transcripts, IFNA2 is unlikely to be the sole mediator of all observed phenotypes; rather, our data support IFNA2 as a critical upstream node that links miR-186-5p depletion to immune-driven β-cell stress, while additional targets may act in parallel to shape the broader β-cell dysfunction observed in prediabetes.

Downstream of IFN-α activation, we observed substantial mitochondrial ROS generation, reduced ATP/ADP ratios, and impaired glucose-stimulated insulin secretion in β-cells under HG+PA conditions. These functional impairments were largely reversed by miR-186-5p mimic, indicating that restoration of miR-186-5p mitigates both innate immune signaling and mitochondrial dysfunction. Since mitochondrial ATP production is a central trigger for insulin secretion [54,55], the ability of miR-186-5p to recover ATP/ADP dynamics highlights its essential role in maintaining β-cell metabolic competence under stress. Ruxolitinib, a JAK inhibitor, also restored STAT1 activation and rescued mitochondrial and insulin secretion phenotypes, further supporting that the detrimental effects of miR-186-5p loss occur through the IFN-α–JAK/STAT pathway.

Our findings indicate that pancreatic β-cells may represent one of several potential contributors to the changes in circulating EV miR-186-5p observed during prediabetes, rather than serving as the sole or dominant source. Under glucolipotoxic conditions in vitro, β-cells exhibited a coordinated reduction in intracellular miR-186-5p and in miR-186-5p levels within EVs released into the culture supernatant, while the β-cell–enriched miRNA miR-375 remained unchanged. This selective pattern supports the notion that miR-186-5p is actively regulated in response to β-cell stress rather than reflecting a nonspecific suppression of EV cargo. Nevertheless, circulating EVs represent a highly heterogeneous population derived from multiple metabolically active tissues, including liver, adipose tissue, and immune cells, all of which are known to undergo stress and inflammatory remodeling during prediabetes. Given the relatively limited β-cell mass and the lack of tissue specificity of miR-186-5p, the present data do not permit direct quantitative attribution of circulating EV miR-186-5p changes to pancreatic β-cells in vivo. Rather, our findings support the interpretation that stressed β-cells constitute a biologically plausible component of a broader, systemic EV miRNA signature associated with early metabolic dysfunction. This view is consistent with the concept of circulating EVs as a “liquid biopsy” that integrates stress signals from multiple tissues [56,57], thereby providing clinically informative biomarkers of early metabolic deterioration, including—but not limited to—β-cell impairment [58,59,60].

The selective reduction in miR-186-5p in EVs during early metabolic stress also raises important biological questions regarding miRNA sorting and β-cell signaling dynamics. One possibility is that diabetic stress alters the machinery responsible for miRNA loading into EVs, such as hnRNPA2B1 or neutral sphingomyelinase 2, resulting in preferential depletion of specific miRNAs like miR-186-5p from both intracellular and EV pools [48,61,62,63,64]. Another explanation is the presence of an autocrine amplification loop in which initial reductions in miR-186-5p permit IFN-α induction, which in turn may further suppress miR-186-5p expression through STAT1-dependent mechanisms [65,66,67]. Such feedback would accelerate β-cell immune activation and mitochondrial stress during prediabetes [50,68]. These insights also highlight therapeutic opportunities: restoring miR-186-5p levels through miRNA mimic delivery could interrupt this feedback loop, while pharmacologic inhibition of IFN-α–JAK/STAT signaling may protect β-cells from early dysfunction. Together, these considerations reinforce the clinical potential of miR-186-5p as both a mechanistic driver and a modifiable therapeutic target in early diabetes.

The present study has several strengths, including the use of two independent human cohorts, rigorous characterization of plasma-derived EVs, integration of clinical associations with mechanistic β-cell experiments, and direct target validation using luciferase reporter assays. Nevertheless, several limitations should be acknowledged. First, the mechanistic analyses were conducted primarily in the murine MIN6 β-cell line. While this model is widely used to investigate β-cell stress signaling, it does not fully recapitulate the complexity and heterogeneity of human pancreatic islets. Therefore, validation of the miR-186-5p–IFN-α regulatory axis in primary human β-cells or isolated human islets will be an important next step to strengthen translational relevance. In addition, the absence of in vivo validation limits direct extrapolation of our mechanistic findings to whole-organism physiology, and future studies using animal models of prediabetes or diabetes are warranted. Second, although reductions in miR-186-5p were observed in both plasma-derived EVs from human subjects and EVs released from stressed β-cells in vitro, direct isolation and quantitative attribution of β-cell–specific EVs within human circulation remain technically challenging. Circulating EVs represent a heterogeneous mixture derived from multiple metabolically active tissues, including liver, adipose tissue, and immune cells, all of which may contribute to systemic EV miRNA profiles under diabetic conditions. Therefore, while our in vitro findings support a biologically plausible contribution of β-cells to circulating EV miR-186-5p reduction, the relative contribution of other tissues cannot be definitively resolved in the current study. Third, although IFNA2 was validated as a direct target of miR-186-5p and provides a plausible mechanistic axis linking miR-186-5p loss to IFN-α–JAK/STAT activation, miR-186-5p is predicted to regulate multiple transcripts that may also contribute to β-cell stress phenotypes. Therefore, the extent to which the observed mitochondrial ROS elevation and insulin secretory defects are mediated exclusively through IFNA2 cannot be fully resolved in the present study. Future work using unbiased transcriptomic/proteomic profiling combined with target-perturbation approaches (e.g., IFNA2 knockdown/overexpression rescue, Ago2-RIP/CLIP-based target validation, and pathway-level analyses) will be necessary to delineate the broader miR-186-5p regulatory network in β-cells.

Overall, our findings identify miR-186-5p as a key regulator of β-cell innate immune activation, mitochondrial oxidative stress, and insulin secretion during early diabetic conditions. The parallel reduction in miR-186-5p in plasma-derived EVs and β-cells suggests that EV miR-186-5p may serve as an early biomarker reflecting underlying β-cell stress in prediabetes. Furthermore, restoring miR-186-5p levels or targeting its downstream IFN-α–JAK/STAT pathway may represent promising therapeutic strategies to preserve β-cell function and prevent progression from prediabetes to T2DM.

## 5. Conclusions

In summary, this study identifies plasma-derived EV miR-186-5p as an early biomarker associated with metabolic deterioration during the transition to prediabetes and supports its functional relevance to β-cell stress responses under diabetic conditions. We demonstrate that miR-186-5p is reduced in circulating EVs in individuals with prediabetes and is selectively downregulated in β-cells exposed to glucolipotoxic stress in vitro. Mechanistically, miR-186-5p directly represses IFNA2, thereby constraining activation of the IFN-α–JAK/STAT–ISG axis, which in turn modulates mitochondrial oxidative stress, cellular energy metabolism, and glucose-stimulated insulin secretion in β-cells. Importantly, while our data support a biologically plausible contribution of stressed β-cells to the observed reduction in circulating EV miR-186-5p, we acknowledge that circulating EV miRNA profiles reflect integrated signals from multiple metabolically active tissues. Thus, EV miR-186-5p should be interpreted as a component of a systemic stress-associated EV signature rather than as a β-cell–exclusive marker. Although IFNA2 represents a validated and mechanistically informative target, miR-186-5p likely coordinates additional downstream pathways that contribute to the broader β-cell stress phenotype. Collectively, our findings position EV miR-186-5p as a non-invasive biomarker reflecting early metabolic stress and highlight the miR-186-5p–IFN-α axis as a potential therapeutic entry point to mitigate β-cell dysfunction and delay progression from prediabetes to T2DM.

## Figures and Tables

**Table 1:** Clinical characteristics of subjects with normoglycemia, prediabetes, and newly diagnosed T2DM in the screening cohort.

Characteristics	Normoglycemia	Prediabetes	T2DM	*p*-Value
Number (Male/Female)	20 (10/10)	20 (8/12)	20 (9/11)	0.137
Age (year)	63.0 ± 4.2	66.2 ± 5.0	59.7 ± 10.6	0.216
BMI (kg/m^2^)	21.8 ± 1.4	22.2 ± 2.3	22.9 ± 1.6	0.079
Waist (cm)	72.9 ± 4.1	77.0 ± 6.0	79.7 ± 6.1	<0.001
HbA1c (%)	5.3 ± 0.2	6.0 ± 0.2	7.8 ± 2.0	<0.001
Fasting blood glucose (mg/dL)	86.9 ± 3.7	105.7 ± 5.3	128.5 ± 25.0	<0.001
Fasting plasma insulin (μIU/mL)	3.4 ± 1.8	6.5 ± 3.4	6.9 ± 4.8	0.003
HOMA-IR	0.7 ± 0.4	1.5 ± 0.8	2.0 ± 1.2	<0.001
Creatinine (mg/dL)	0.7 ± 0.1	0.8 ± 0.1	0.7 ± 0.2	0.634
eGFR (mL/min/1.73 m^2^)	93.5 ± 16.2	83.1 ± 17.0	94.0 ± 22.5	0.909
LDL (mg/dL)	117.0 ± 30.5	118.4 ± 31.8	113.9 ± 44.3	0.788
AST (IU/L)	23.1 ± 5.4	23.6 ± 5.7	25.4 ± 7.1	0.234
ALT (IU/L)	19.1 ± 6.8	20.1 ± 8.9	20.8 ± 12.5	0.413
SBP (mmHg)	118.6 ± 17.6	127.9 ± 14.3	120.3 ± 13.3	0.723
DBP (mmHg)	75.2 ± 7.8	76.6 ± 8.3	71.8 ± 11.4	0.269

Data are presented as counts or as mean values with corresponding standard deviations. Statistical significance was defined at a threshold of *p* < 0.05. T2DM, type 2 diabetes; BMI, body mass index; HbA1c, hemoglobin A1c; HOMA-IR, Homeostatic model assessment of insulin resistance; eGFR, estimated glomerular filtration rate; LDL, low-density lipoprotein cholesterol; AST, aspartate aminotransferase; ALT, alanine aminotransferase; SBP, systolic blood pressure; DBP, diastolic blood pressure.

**Table 2:** Plasma-derived EV miRNAs showing >2-fold differential expression across glycemic groups identified by small RNA sequencing.

Mature hsa-miRNA	PreDM/NG	T2DM/PreDM	T2DM/NG	*p*-Value
hsa-miR-10b-5p	4.60	5.98	6.98	0.026
hsa-miR-19a-3p	−2.28	−2.94	−6.68	0.001
hsa-miR-184	3.64	6.44	7.67	0.013
hsa-miR-186-5p	−2.62	−2.35	−6.15	0.001
hsa-miR-326	−5.09	−2.38	−12.13	0.001
hsa-miR-376c-3p	10.49	6.80	5.96	0.008
hsa-miR-377-3p	10.67	4.10	3.83	0.003
hsa-miR-382-5p	−2.34	−2.53	−5.94	0.003
hsa-miR-485-3p	14.72	8.90	8.43	0.001
hsa-miR-1290	2.46	4.92	12.08	0.000
hsa-miR-1307-3p	−2.68	−2.39	−6.39	0.002
hsa-miR-4286	−3.50	−2.84	−9.94	0.000

Statistical significance was defined as *p* < 0.05. Hsa, Homo sapiens; miR, microRNA; NG, normoglycemia; PreDM, prediabetes; T2DM, type 2 diabetes.

**Table 3:** Clinical characteristics of subjects included in the independent validation cohort.

Characteristics	Normoglycemia	Prediabetes	T2DM	*p*-Value
Number (Male/Female)	50 (25/25)	50 (25/25)	50 (26/24)	0.329
Age (year)	59.3 ± 5.72	63.4 ± 7.31	64.8 ± 8.14	0.092
BMI (kg/m^2^)	55.0 ± 9.01	55.4 ± 8.59	57.6 ± 8.68	0.761
Waist (cm)	22.3 ± 1.73	23.0 ± 1.69	22.8 ± 1.74	0.628
HbA1c (%)	5.3 ± 0.21	5.9 ± 0.15	7.5 ± 1.39	<0.001
Fasting blood glucose (mg/dL)	89.9 ± 5.27	109.7 ± 6.60	138.7 ± 44.14	<0.001
Fasting plasma insulin (μIU/mL)	3.2 ± 1.24	8.3 ± 3.52	8.7 ± 3.98	0.011
HOMA-IR	0.7 ± 0.21	1.7 ± 1.09	3.5 ± 7.59	<0.001
Creatinine (mg/dL)	0.7 ± 0.26	0.7 ± 0.15	0.7 ± 0.21	0.723
eGFR (mL/min/1.73 m^2^)	96.5 ± 16.63	95.2 ± 15.38	96.0 ± 17.90	0.967
LDL (mg/dL)	111.8 ± 32.71	116.4 ± 30.44	118.5 ± 38.43	0.672
AST (IU/L)	22.9 ± 5.17	23.2 ± 5.65	24.3 ± 6.73	0.438
ALT (IU/L)	16.8 ± 5.62	19.6 ± 7.54	20.4 ± 8.42	0.264
SBP (mmHg)	118.7 ± 15.73	121.8 ± 19.42	129.1 ± 21.44	0.103
DBP (mmHg)	79.2 ± 6.91	76.4 ± 8.15	72.9 ± 10.12	0.574

Data are presented as counts or as mean values with corresponding standard deviations. Statistical significance was defined at a threshold of *p* < 0.05. T2DM, type 2 diabetes; BMI, body mass index; HbA1c, hemoglobin A1c; HOMA-IR, Homeostatic model assessment of insulin resistance; eGFR, estimated glomerular filtration rate; LDL, low-density lipoprotein cholesterol; AST, aspartate aminotransferase; ALT, alanine aminotransferase; SBP, systolic blood pressure; DBP, diastolic blood pressure.

**Table 4:** Pearson correlation analysis between metabolic parameters and validated EV miRNAs.

		miR-19a-3p	miR-186-5p	miR-326	miR-382-5p	miR-485-3p	miR-1307-3p
FBG	Correlation R	−0.211	−0.214	−0.217	−0.214	−0.249	−0.202
	*p*-value	0.011	0.011	0.019	0.027	0.002	0.015
HbA1C	Correlation R	−0.208	−0.188	−0.212	−0.223	−0.257	−0.205
	*p*-value	0.012	0.025	0.022	0.041	0.002	0.013
Insulin	Correlation R	−0.183	−0.201	−0.138	−0.153	−0.223	−0.178
	*p*-value	0.068	0.039	0.057	0.052	0.012	0.054
HOMA-IR	Correlation R	−0.206	−0.219	−0.214	−0.209	−0.236	−0.207
	*p*-value	0.033	0.029	0.016	0.035	0.003	0.035

A value of *p* < 0.05 was considered statistically significant. FBG, fasting blood glucose; HbA1c, hemoglobin A1c; HOMA-IR, Homeostatic model assessment of insulin resistance; miR, microRNA.

**Table 5:** Multivariate logistic regression analyses identifying EV miRNA predictors of prediabetes.

	**Regression** **Coefficient**	**Standard** **Error**	**Wald**	**Degrees of Freedom**	***p*** **-Value**	**Estimated Odd Ratio**	**95% Confidence Interval**
miR-19a-3p	−0.219	0.289	0.573	1	0.449	0.804	0.456–1.415
miR-186-5p	−1.987	0.783	6.438	1	0.011	0.137	0.030–0.636
miR-326	0.066	0.596	0.012	1	0.912	1.068	0.332–3.438
miR-382-5p	−0.397	0.292	1.85	1	0.174	0.672	0.380–1.191
miR-485-3p	−0.549	0.316	3.005	1	0.083	0.578	0.311–1.074
miR-1307-3p	−0.207	0.517	0.16	1	0.689	0.813	0.295–2.239
(constant)	1.052	0.48	4.799	1	0.028	2.862	

Statistical significance was defined as *p* < 0.05. miR, microRNA.

## Data Availability

The original contributions presented in the study are included in the article/Supplementary Material. The small RNA sequencing data generated in this study have been deposited in a public repository and are available under the accession number PRJNA1406448. Further inquiries can be directed to the corresponding author.

## Supplementary Materials

The following supporting information can be downloaded at: https://www.mdpi.com/article/10.3390/antiox15020150/s1. Figure S1: Volcano plots of plasma-derived EV miRNAs based on raw *p*-values. Figure S2: Intracellular expression of six candidate EV miRNAs in MIN6 β-cells under high glucose and palmitate treatment.

## Abbreviations

The following abbreviations are used in this manuscript:

EV	Extracellular vesicle
IFN	Type I interferon
ISG	Interferon-stimulated gene
miRNA	MicroRNA
ROS	Reactive oxygen species
T2DM	Type 2 diabetes mellitus
