# Supplementary material for: Plasma EV miR-186-5p as an Early Biomarker and Regulator of IFN-α-Mediated Oxidative and β-Cell Dysfunction in Prediabetes
Source: Antioxidants (Basel). 2026 Jan 23;15(2):150. doi: 10.3390/antiox15020150 (PMC12937789; doi:10.3390/antiox15020150)
Supplement: Supplementary file 1 [file antioxidants-15-00150-s001.zip › Supplementary Figures.pdf]

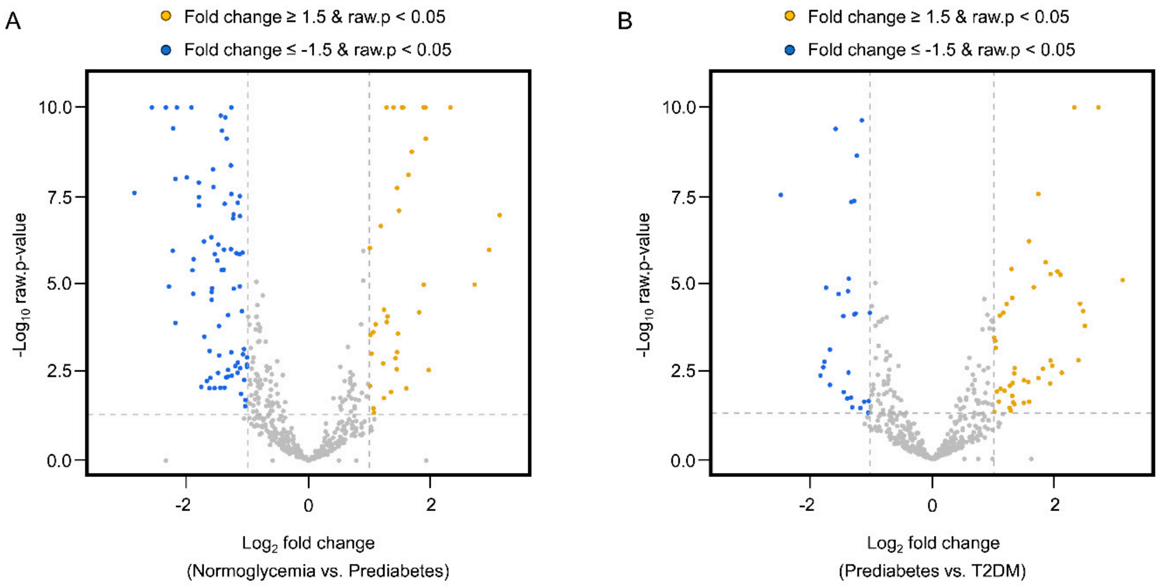

Supplementary Figure S1. Volcano plots of plasma-derived EV miRNAs based on raw p-values. (A) Volcano plot showing differentially expressed EV miRNAs in prediabetes compared with normoglycemia based on unadjusted (raw) p-values. (B) Volcano plot showing differentially expressed EV miRNAs in T2DM compared with prediabetes based on unadjusted (raw) p-values. These plots are provided for reference, while all candidate miRNAs selected for downstream validation were identified based on FDR-adjusted statistical significance as shown in Figure 2. T2DM, type 2 diabetes.

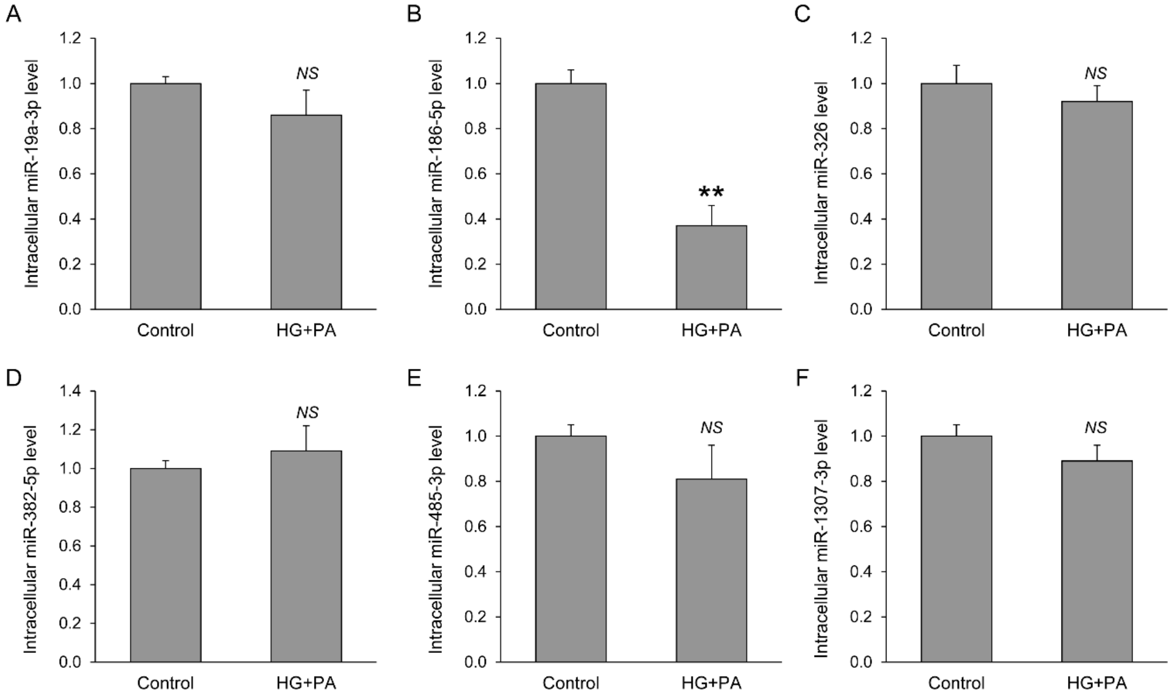

Supplementary Figure S2. Intracellular expression of six candidate EV miRNAs in MIN6 β-cells under high glucose and palmitate treatment. qRT-PCR analysis of (A) miR-19a-3p, (B) miR-186-5p, (C) miR-326, (D) miR-382-5p, (E) miR-485-3p, and (F) miR-1307-3p in MIN6 cells treated with either control medium or HG+PA. miRNA expression levels were normalized to U6 snRNA and are presented as mean ± SEM from three independent experiments (n = 5). $^{**}p < 0.01$ vs. control. HG+PA, high glucose and palmitate; NS, not significantly.
